# Hierarchically Porous Hollow TiO_2_ Nanofibers Coupled with Fluorescence-Tuned Graphene Quantum Dots for Efficient Visible-Light Photocatalysis

**DOI:** 10.3390/molecules31091430

**Published:** 2026-04-26

**Authors:** Weitao Li, Zeyun Dong, Zhengyu Zhang, Luoman Zhang, Qizhe Wang, Shang Li, Shuai Li, Lei Wang, Jialin Liu

**Affiliations:** 1 Textile and Garment Industry of Research Institute, Zhongyuan University of Technology, Zhengzhou 450007, China; liweitao@zut.edu.cn (W.L.); 2023117759@zut.edu.cn (Z.D.); z1543086543@163.com (L.Z.); wangqizhe@zut.edu.cn (Q.W.); lishang@zut.edu.cn (S.L.); 2024117032@zut.edu.cn (S.L.); 2 Department of Environment, Yangtze Delta Region Institute of Tsinghua University, Jiaxing 314006, China; zhangzyever@gmail.com

**Keywords:** graphene quantum dots, TiO_2_ nanofibers, heterojunction, fluorescence redshift, photocatalytic degradation

## Abstract

Industrial dye wastewater poses serious environmental and health risks, demanding sustainable remediation strategies. Here, hierarchically porous hollow TiO_2_ nanofibers (HNFTis) were fabricated and combined with blue, green, and orange graphene quantum dots (b-GQDs, g-GQDs, o-GQDs) to form heterojunction photocatalysts. Progressive fluorescence redshift of GQDs, induced by surface functionalization and S/B doping, narrows the bandgap and enhances visible-light absorption. Among the composites, 0.5 wt% o-GQDs/HNFTi exhibited the highest photocurrent and lowest charge-transfer resistance and degraded 99.5% of Methylene blue under visible light within 2 h, outperforming pristine HNFTi (77.7%). The synergistic effect of TiO_2_ structural engineering and GQD fluorescence tuning demonstrates an effective strategy for designing high-performance photocatalysts for sustainable wastewater treatment.

## 1. Introduction

Industrial dye wastewater, especially containing Methylene blue (MB), poses severe ecological and health risks due to its toxicity and resistance to conventional treatments. It contains highly toxic and biorefractory organic dyes with potential carcinogenic, teratogenic and mutagenic risks. These pollutants severely damage aquatic ecosystems and threaten human health via bioaccumulation [1,2,3,4,5,6,7,8,9]. Photocatalysis offers a sustainable route for complete mineralization of such contaminants using solar energy. When irradiated by light with energy beyond its bandgap, the photocatalyst generates electron–hole pairs. Electrons in the conduction band reduce O_2_ to superoxide radical (·O_2_^−^), whereas holes in the valence band oxidize H_2_O or hydroxide ion (OH^−^) to hydroxyl radical (·OH). As strong oxidants, these reactive oxygen species attack organic dye molecules, destroying their conjugated structures and gradually mineralizing them into CO_2_, H_2_O and other harmless small molecules [10,11,12,13]. Titanium dioxide (TiO_2_) is widely explored as a photocatalyst for its stability and low cost, yet its wide bandgap (3.2 eV) restricts absorption to UV light, and rapid electron–hole recombination limits efficiency under visible light [14,15,16,17,18,19,20,21,22]. Structural engineering and heterojunction formation have emerged as effective strategies to overcome these limitations. Electrospinning is a simple and efficient technique to fabricate micro/nanofibers. Under a high-voltage electric field, charged polymer solution is stretched from Taylor cone into a fine jet. With solvent evaporation, continuous nanofibers are formed and deposited. The prepared nanofibrous membrane has a large specific surface area and porous structure, which is beneficial for mass transfer and light absorption in photocatalysis. Hierarchically porous hollow TiO_2_ nanofibers (HNFTis), fabricated via electrospinning, provide high surface area, interconnected porosity, and hollow channels, enhancing active site availability, light scattering, and charge transport [23,24,25,26,27,28,29,30,31]. Meanwhile, graphene quantum dots (GQDs) act as photosensitizers with tunable fluorescence, strong upconversion emission, and efficient electron-accepting capability [32,33,34,35,36]. Surface functionalization and heteroatom doping (using sulfur (S) or boron (B)) induce a progressive fluorescence redshift in GQDs, narrowing the bandgap and extending visible-light absorption [37]. When integrated with HNFTi, GQDs facilitate charge separation and boost solar energy utilization [38].

Herein, blue (b-GQDs), green (g-GQDs), and orange (o-GQDs) quantum dots were hydrothermally synthesized and anchored onto HNFTi to form heterojunction photocatalysts. Comprehensive studies on optical properties, charge-transfer dynamics, and MB degradation reveal that the combination of TiO_2_ structural modulation and GQD fluorescence tuning synergistically enhances visible-light-driven photocatalysis, providing a rational design strategy for high-performance, sustainable photocatalysts.

## 2. Result

### 2.1. Structural and Optical Properties of b-, g-, and o-GQDs

**Scheme 1:**
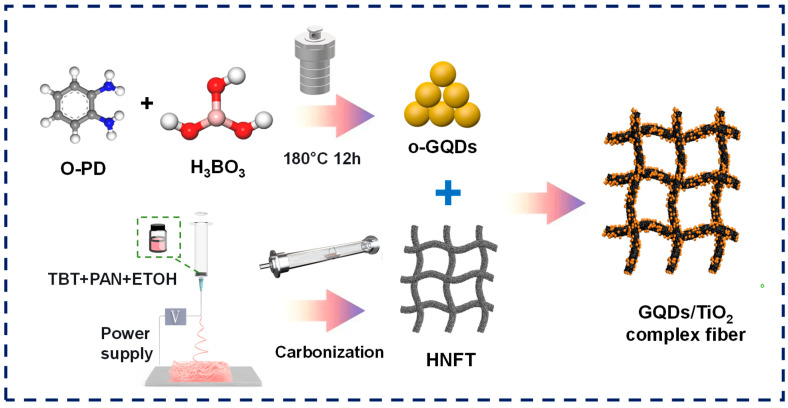
The procedure used to fabricate the o-GQDs/TiO_2_ heterojunction.

In this study, three emissive graphene quantum dots (i.e., blue-, green-, and orange-emitting GQDs) were hydrothermally synthesized. TiO_2_, hollow fibers with a high surface area (45.74 m^2^ g^−1^) (Brunauer–Emmett–Teller (BET) surface areas and Barrett–Joyner–Halenda (BJH) pore size distributions were obtained from N_2_ adsorption–desorption isotherms recorded at 77 K on a Micromeritics ASAP 2460 surface area analyzer) and abundant active sites were fabricated by electrospinning. Hollow TiO_2_ nanofibers (HNFTis) were further integrated with the as-prepared GQDs to establish novel heterostructured photocatalysts, as shown in Scheme 1.

**Figure 1:**
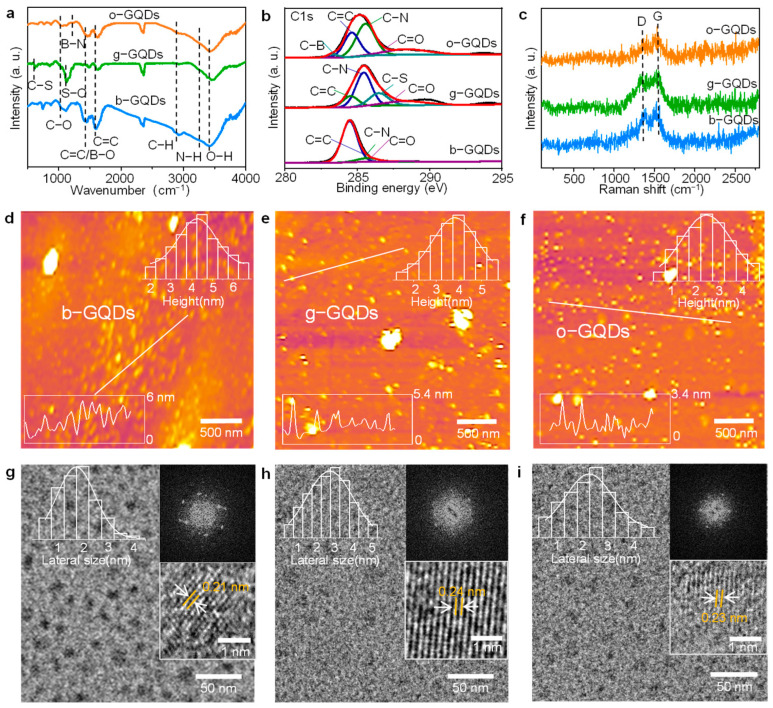
(**a**) FTIR spectra, (**b**) XPS C 1s spectra, and (**c**) Raman spectra of b-, g-, and o-GQDs. (**d**–**f**) AFM images. (**g**–**i**) TEM images, with insets showing Fourier transform patterns and HRTEM images, corresponding to b-, g-, and o-GQDs.

The surface chemistry of the GQDs was analyzed by FTIR (corresponding to the fast Fourier transform analysis of the HRTEM images) and XPS (Figure 1a,b and Figures S1–S3). All samples (b-, g-, o-GQDs) exhibited C–C, C=C, C–H, and N–H vibrations, indicating hydroxyl (-OH) and amine (-NH_2_) functionalization, along with weak carboxyl (–COOH) groups [39]. Additional C–S/S=O bands in g-GQDs and B–O/B–N signals in o-GQDs confirmed successful S and B doping [40], respectively. XPS further revealed that from b- to o-GQDs, electron-donating groups (–OH, –NH_2_) decreased while –COOH increased (0.99% → 10.91%), consistent with a fluorescence redshift induced by electron-withdrawing surface functionalities [41,42]. XRD patterns showed a broad peak near 26°, corresponding to the (002) plane of graphitic carbon (Figure S4). Raman spectra (Figure 1c) exhibited D and G bands, the IG/ID value represents the graphitization degree of carbon nanomaterials: a higher IG/ID indicates fewer defects and higher structural order, with IG/ID ratios increasing from 1.03 (b-GQDs) to 1.23 (o-GQDs), indicating enhanced graphitization and structural ordering, which reduces the bandgap and contributes to the redshift [43]. AFM (Figure 1d–f) revealed thicknesses of 3.65 nm and TEM (Figure 1g–i) showed quasi-spherical particles with diameters of 2.83 nm, while HRTEM confirmed lattice fringes of 0.21–0.24 nm indexed to graphene (100) planes. Slight lattice expansion from b- to o-GQDs is attributed to heteroatom doping and functionalization, perturbing the π-conjugated framework [44] (Figure S5).

**Figure 2:**
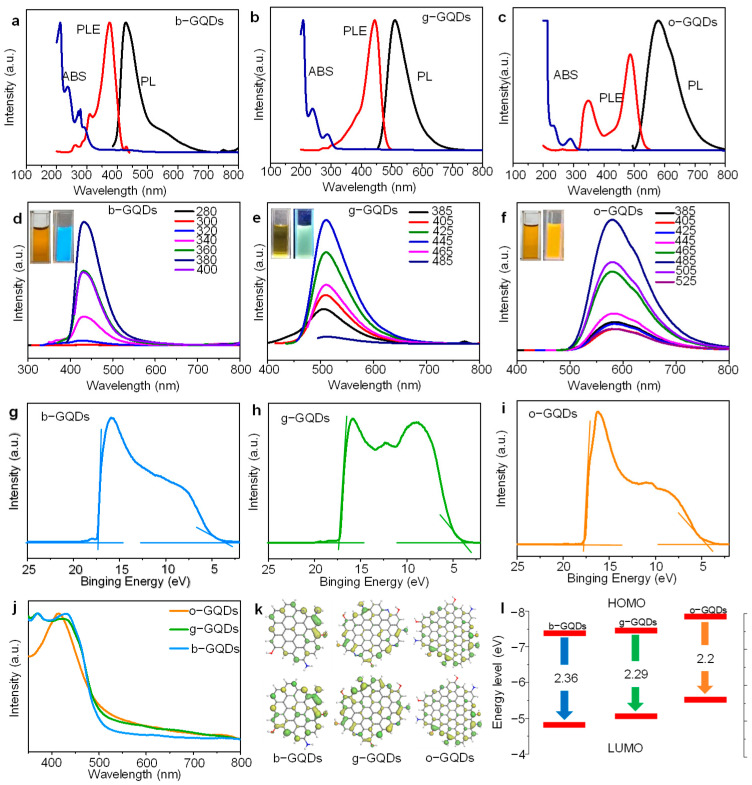
(**a**–**c**) UV-vis absorption, PL, and PLE spectra of b-, g-, and o-GQDs. (**d**) UPS spectra of b-, g-, and o-GQDs. (**e**) Fitting curves of maximum excitation wavelength and absorption peak. (**f**) Correlation between particle size of GQDs and their optimal emission wavelength (**g**) Calculated optical bandgaps. (**h**–**l**) Time-dependent density functional theory calculation results: calculated HOMO-LUMO energy level orbital distributions.

UV-vis spectra (Figure 2a–c) displayed characteristic π-π* (C=C) and n-π* (C=O) transitions, with absorption edges redshifting from b- to o-GQDs (236–292 nm → 238–290 nm) (Figure S6) [45,46]. The optimal excitation/emission pairs were 380/430 nm (b-GQDs), 445/510 nm (g-GQDs), and 485/580 nm (o-GQDs), consistent with observed fluorescence (Figure S7). UPS spectra (Figure 2d) revealed variations in electronic structures. Figure 2e showed fitting curves of maximum excitation wavelength and absorption peak. A clear correlation between lateral size and emission wavelength was observed in Figure 2f [47]. The optical bandgaps estimated from DRS (Figure 2g) decreased from 2.36 eV (b-GQDs) and 2.29 eV (g-GQDs) to 2.20 eV (o-GQDs) (Table S1). The HOMO levels were determined by UPS (Figure 2h), while the corresponding LUMO levels were deduced by subtracting the optical bandgap from the HOMO values (Figure 2i). Structural models (Figure 2h) highlight the increasing π-conjugated domains and evolution of surface functionalities, which stabilize the electronic structure, narrow the bandgap, and enhance visible-light absorption and charge-separation efficiency, with o-GQDs exhibiting the most favorable properties.

### 2.2. Morphology and Photocatalytic Properties of Different TiO_2_ Nanostructures

**Figure 3:**
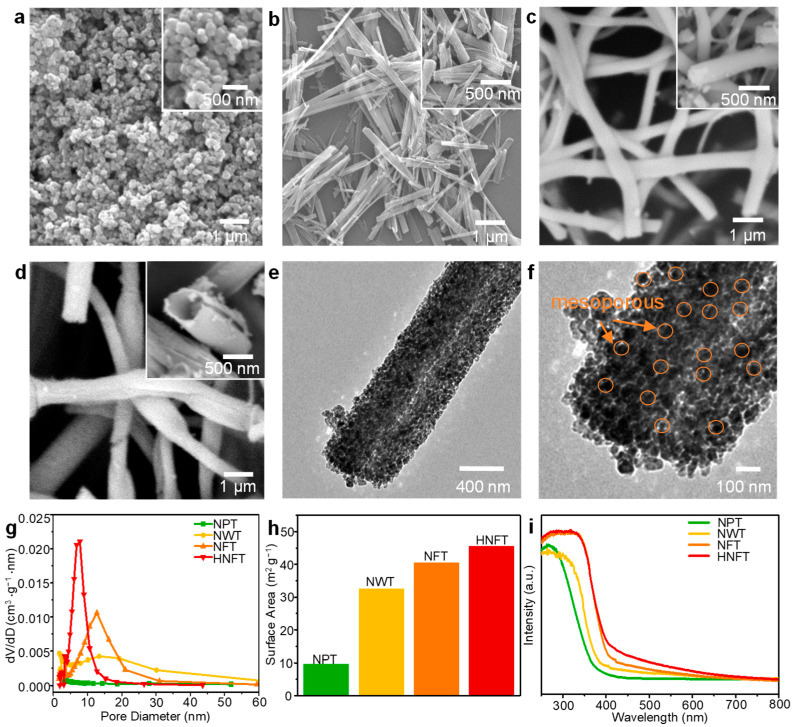
(**a**–**d**) SEM images of NPTi, NWTi, NFTi, and HNFTi. (**e**,**f**) TEM images of HNFTi. (**g**) Pore size distribution curves, (**h**) BET surface area, and (**i**) UV-vis DRS.

SEM images (Figure 3a–d) revealed distinct morphologies: NPTi as aggregated nanoparticles, NWTi as nanowires, NFTi as dispersed fibers, and HNFTi as hollow fibers with rough surfaces and pores confirmed by TEM; the yellow circles highlight distinct regions on the fiber surface that exhibit pronounced irregular roughness and porous morphological features (Figure 3e,f). Pore size distribution curves (Figure 3g) further confirm the uniform mesoporous structure of HNFTi. The hollow structure of HNFTi arises from phase separation during electrospinning and calcination. BET analysis (Figure 3h) showed type IV isotherms with H_2_/H_3_ hysteresis for NFTi/HNFTi (Figure S7), indicating uniform mesopores [48]. HNFTi exhibited the highest surface area (45.74 m^2^ g^−1^) and pore volume (0.13 cm^3^/g), providing abundant active sites and enhanced MB adsorption [49]. DRS indicated bandgaps of 3.17 (NPTi), 2.93 (NWTi), 2.81 (NFTi), and 2.69 eV (HNFTi) (Figure S8), where the narrower bandgap of HNFTi facilitates visible-light absorption and charge separation (Figure 3i). Furthermore, all samples exhibit typical type IV isotherms with H3 hysteresis loops, confirming the mesoporous structure of the materials. The adsorption capacity increases slightly with GQD loading, which is consistent with the enhanced BET surface area (0.5b-GQDs/HNFTi (47.22 m^2^ g^−1^), 0.5g-GQDs/HNFTi (48.15 m^2^ g^−1^) and 0.5o-GQDs/HNFTi (49.36 m^2^ g^−1^)), indicating that GQD modification does not destroy the porous structure but provides additional active sites (Figure S9).

### 2.3. Characterization of GQD/HNFTi Heterojunctions

**Figure 4:**
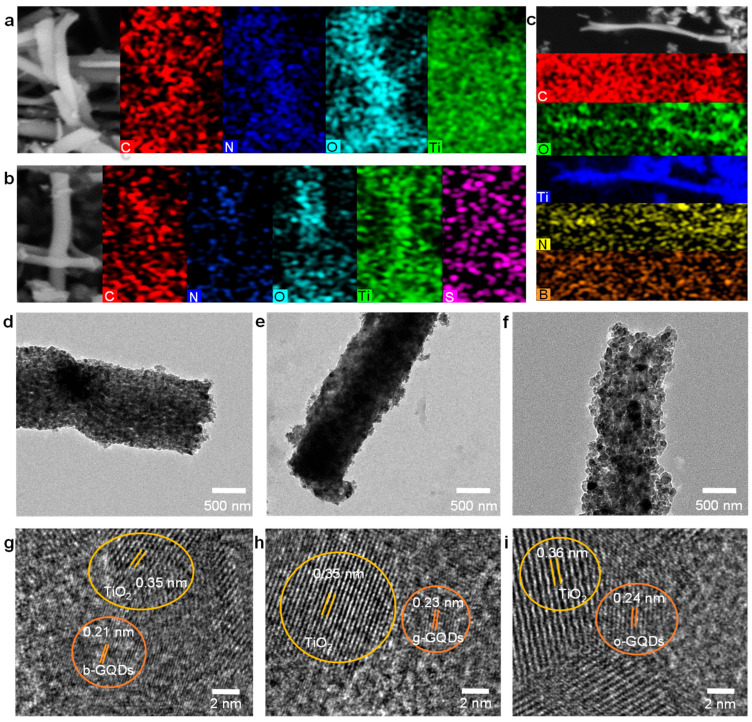
(**a**–**c**) SEM and EDS elemental mapping of 0.5b-GQDs/HNFTi, 0.5g-GQDs/HNFTi, and 0.5o-GQDs/HNFTi. (**d**–**f**) TEM images, and (**g**–**i**) HRTEM images of 0.5b-GQDs/HNFTi, 0.5g-GQDs/HNFTi, and 0.5o-GQDs/HNFTi.

To validate GQD/TiO_2_ heterojunction formation, characterization analysis was subsequently performed on the composite samples. SEM and EDS mapping (Figure 4a–c) confirmed uniform deposition of C, N, S, or B on TiO_2_ fibers, verifying heterojunction formation. TEM/HRTEM (Figure 4d–i) showed preserved fibrous morphology and well-resolved lattice fringes for both TiO_2_ and GQDs, indicating that loading does not disrupt crystallinity. XRD patterns (Figure S10a) displayed typical anatase peaks with no shifts, suggesting GQDs reside on the fiber surface rather than the lattice. FTIR (Figure S10b) confirmed C=C stretching from GQDs, while Ti–O–Ti vibrations remained unchanged. Raman spectra (Figure S10c) showed anatase modes along with D/G bands of GQDs, supporting successful heterojunction construction.

### 2.4. Photocatalytic Performance of GQDs/HNFTi

**Figure 5:**
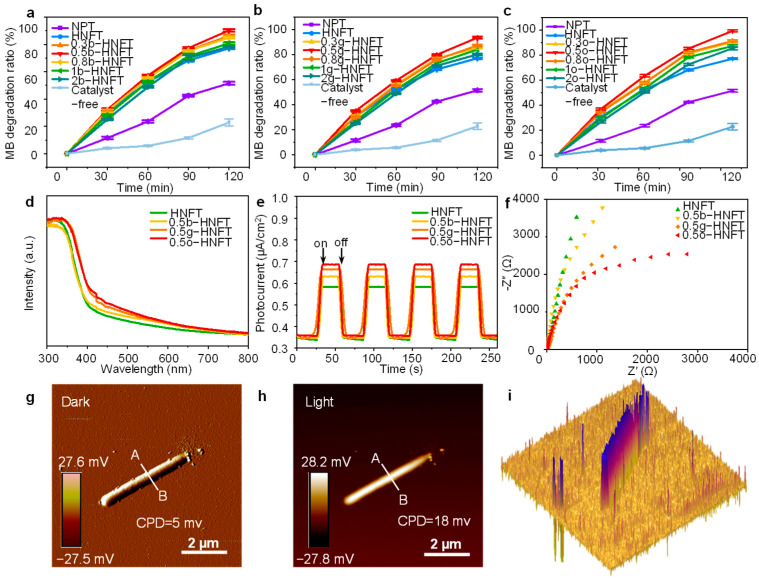
MB degradation efficiency of pure HNFTi and GQD-modified heterojunction photocatalysts with different contents of b-GQDs (**a**), g-GQDs (**b**), b-GQDs, and (**c**) o-GQDs under visible light. (**d**) UV-vis DRS, (**e**) Transient photocurrent responses, and (**f**) EIS spectra of different photocatalysts. (**g**–**i**) KPFM surface potential mappings of 0.5o-GQDs/HNFTi in darkness, under light, and the corresponding 3D image.

To obtain the best photocatalytic performance, the optimal GQD loading content, initial MB concentration, and catalyst dosage were carefully optimized. A series of GQD/HNFTi composites with different GQD loadings (0.25, 0.5, 0.75, and 1.0 wt%) were prepared and evaluated (Figure 5a–c). The photocatalytic activity first increased and then decreased with increasing GQD content. The highest efficiency was achieved at 0.5 wt% GQD loading. Lower loading could not provide sufficient heterojunction interfaces to boost charge separation. Excessive GQDs tended to aggregate on the fiber surface, blocking active sites, shielding light absorption, and acting as charge recombination centers, thus reducing the photocatalytic efficiency. Therefore, 0.5 wt% was determined as the optimal GQD loading for all composite samples (Figure S11). The initial concentration of MB substrate was also optimized. A moderate initial concentration of 10 mg·L^−1^ was selected to ensure obvious and reliable concentration changes during the photocatalytic reaction, which facilitated accurate quantitative detection via UV-vis spectroscopy and reasonable kinetic analysis.

The mass of catalyst used was fixed at 20 mg to ensure sufficient active sites and uniform light absorption. Prior to irradiation, the suspension was stirred in the dark for 30 min to reach adsorption desorption equilibrium. The dark-adsorption curve (Figure S12) confirms that adsorption contributes little to MB removal, and the efficiency loss mainly originates from photocatalytic degradation. The blank experiment without catalyst rules out direct self-photolysis of MB (Figure 5a–c). Photocatalytic degradation curves demonstrated that all GQD-modified samples exhibited higher activity than pure HNFTi (Figure S13). The optimal 0.5 wt% GQDs loaded samples showed MB degradation efficiencies of 90.1% for 0.5b-GQDs/HNFTi, 94.3% for 0.5g-GQDs/HNFTi, and 99.5% for 0.5o-GQDs/HNFTi. UV-vis DRS (Figure 5d) confirmed redshifted absorption edges and narrowed bandgaps (HNFTi 2.60 eV → 0.5o-HNFTi 2.46 eV), enhancing visible-light absorption and charge separation [50]. Transient photo current (Figure 5e) and EIS (Figure 5f) analyses indicated superior carrier transport for composites, especially 0.5o-HNFTi [51]. KPFM measurements (Figure 5g–i) showed a contact potential difference (CPD) increase under illumination, confirming efficient electron–hole pair separation at the heterojunction interface [52,53].

### 2.5. Degradation Pathway and Mechanism of GQDs/HNFTi

**Figure 6:**
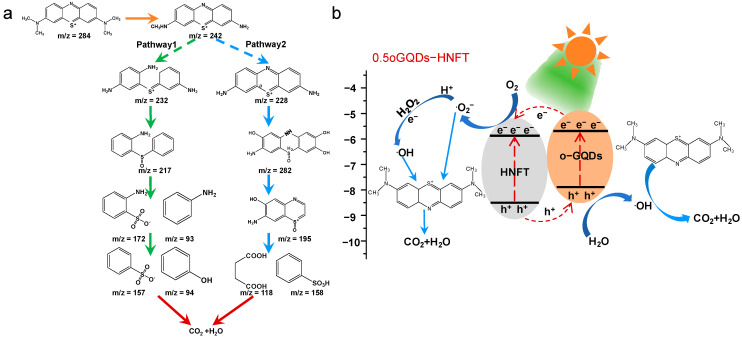
(**a**) Proposed MB degradation pathways. (**b**) Mechanism diagram of 0.5o-HNFTi photocatalytic.

Under visible light, electrons in HNFTi and o-GQDs are excited to their conduction bands, while holes remain in the valence bands. Band alignment drives electron transfer from o-GQDs to HNFTi and hole migration from HNFTi to o-GQDs, suppressing recombination. Photogenerated electrons reduce oxygen (O_2_) to superoxide anion (·O_2_^−^), which converts to hydrogen peroxide (H_2_O_2_) and hydroxyl radical (·OH); holes oxidize H_2_O or hydroxide (OH^−^) to ·OH. These radicals drive stepwise MB degradation, ultimately producing CO_2_ and H_2_O. To clarify the detailed degradation process, LC-MS was employed to identify the intermediates generated during MB photodegradation, and the results are displayed in Figure 6a. A series of typical molecular ion peaks were clearly detected, revealing the stepwise and progressive decomposition pathway of MB. Initially, MB undergoes continuous N-demethylation, during which the methyl groups attached to the nitrogen atoms are gradually removed, producing a variety of demethylated derivatives. Subsequently, the conjugated chromophore structure is destroyed, followed by aromatic ring cleavage to generate small molecular organic intermediates. Meanwhile, sulfur oxidation takes place, converting the sulfur-containing thiazine ring into oxidized fragments. Under the continuous attack of reactive oxygen species, these intermediates are further oxidized into short-chain aliphatic compounds and eventually mineralized into CO_2_ and H_2_O.The identified intermediates and proposed degradation pathways are highly consistent with the photocatalytic mechanism of the GQDs/TiO_2_ heterojunction system (Figure 6b) [54,55].

## 3. Materials and Methods

### 3.1. Materials

O-phenylenediamine (Shanghai Sinopharm Chemical Reagent Co., Ltd., Shanghai, China) (C_6_H_8_N_2_, o-PD, CAS No.: 95-54-5, *M*_r_: 108.14), boric acid (Shanghai Sinopharm Chemical Reagent Co., Ltd., Shanghai, China) (H_3_BO_3_, BA, CAS No.: 10043-35-3, *M*_r_: 61.83), and sulfanilic acid (Shanghai Sinopharm Chemical Reagent Co., Ltd., Shanghai, China) (C_6_H_7_NO_3_S, SA, CAS No.: 121-57-3, *M*_r_: 173.19). Citric acid (Adamas, Beijing, China) (C_6_H_8_O_7_, CA, CAS No.: 77-92-9, *M*_r_: 192.12). Sodium hydroxide (Shanghai Sinopharm Chemical Reagent Co., Ltd., Shanghai, China) (NaOH, CAS No.: 1310-73-3, *M*_r_: 39.998) and anhydrous ethanol (Shanghai Sinopharm Chemical Reagent Co., Ltd., Shanghai, China) (C_2_H_5_OH, ETOH, CAS No.: 64-17-5, *M*_r_: 46.07). Anatase TiO_2_ powder (nanoparticles) (Sigma-Aldrich, St. Louis, MO, USA) (TiO_2_, CAS No.: 13463-67-7, *M*_r_: 79.87). Tetrabutyl titanate (Tianjin Komeo Chemical Reagent Co., Ltd., Tianjin, China) (C_16_H_36_O_4_Ti, TBT, CAS No.: 5593-70-4, *M*_r_: 340.36). Glacial acetic acid (Shanghai Aladdin Biochemical Technology Co., Ltd., Shanghai, China) (C_2_H_4_O_2_, CAS No.: 64-19-7, *M*_r_: 60.05). *N*,*N*-dimethylformamide (Tianjin Zhiyuan Chemical Reagent Co., Ltd., Tianjin, China) (C_3_H_7_NO, DMF, CAS No.: 68-12-2, *M*_r_: 73.09). Methylene blue (Shanghai Titan Technology Co., Ltd., Shanghai, China) (C_16_H_18_ClN_3_S, MB, CAS No.: 61-73-4, *M*_r_: 319.85), polyacrylonitrile (Shanghai Titan Technology Co., Ltd., Shanghai, China) ((C_3_H_3_N)n, PAN, CAS No.: 25014-41-9, *M*_r_: 150,000), and quinine sulfate (Shanghai Titan Technology Co., Ltd., Shanghai, China) (C_20_H_24_N_2_O_2_·H_2_SO_4_, CAS No.: 6119-70-2, *M*_r_: 396.43). All reagents were of analytical grade and used as received without further purification. Deionized water was obtained from a Master Series water purification system (Shanghai Hitech Instruments Co., Ltd., Shanghai, China).

### 3.2. Synthesis of Blue-Graphene Quantum Dots (b-GQDs), Green-Graphene Quantum Dots (g-GQDs), Orange-Graphene Quantum Dots (o-GQDs)

Blue-, green-, and orange-emissive graphene quantum dots (b-GQDs, g-GQDs, and o-GQDs) were synthesized via a hydrothermal method. Typically, o-PD (0.1 g) and CA (0.05 g) were dissolved in 10 mL of deionized water and ultrasonicated for 30 min to form a homogeneous solution. The solution was transferred to a Teflon-lined stainless-steel autoclave (Yanzheng Experimental Instrument Co., Ltd., Zhengzhou, China) and heated at 180 °C for 12 h. After cooling to room temperature, the product was filtered through a 0.22 μm microporous membrane (Jinteng Experimental Equipment Co., Ltd., Tianjin, China) to furnish b-GQDs. g-GQDs and o-GQDs were prepared using the same protocol, except that CA was replaced with SA and BA, respectively. Quantum yields were determined using quinine sulfate (0.1 mol L^−1^ H_2_SO_4_, Φ = 0.546) as a standard according to the comparative approach.

### 3.3. Hollow-Structured TiO_2_ Nanofibers (HNFTi)

TiO_2_ nanofibers were prepared via electrospinning. PAN (2 g), glacial acetic acid (0.5 g), and TBT (4 g) were dissolved in ethanol (20 mL) under vigorous stirring to obtain a clear precursor solution. Electrospinning was carried out using a single-nozzle electrospinning apparatus (Kato Tech Co., Ltd., Kyoto, Japan) under the following conditions: distance, 20 cm; voltage, +20 kV/−1.5 kV; feed rate, 1 cm h^−1^. After 4 h of spinning, the nanofiber mats were pre-oxidized at 200 °C for 2 h and subsequently calcined at 500 °C for 2 h in a muffle furnace (Yingshang Electric Furnace Co., Ltd., Shanghai, China) to afford HNFTi.

### 3.4. Fabrication of GQD/TiO_2_ Composites

HNFTi (0.2 g) was dispersed in aqueous solutions containing different loadings of each type of GQD (0, 0.3, 0.5, 0.8, 1, and 2 wt%, relative to HNFTi). Each mixture was magnetically stirred using a digital magnetic stirrer (IKA Works Co., Ltd., Staufen, Germany) and gently heated at 50 °C using a ceramic heating plate (IKA Works Co., Ltd., Staufen, Germany) to evaporate excess water. The resulting solids were dried at 60 °C in a forced-air drying oven (Shanghai Yiheng Scientific Instrument Co., Ltd., Shanghai, China) to obtain GQD/TiO_2_ composites, denoted as b-GQDs/HNFTi, g-GQDs/HNFTi, and o-GQDs/HNFTi, respectively.

### 3.5. Characterization

The morphology of TiO_2_ nanostructures was examined by scanning electron microscopy (SEM, Phenom Pure, Thermo Fisher Scientific, Waltham, MA, USA) and transmission electron microscopyTEM, JEM-2100F, JEOL Ltd., (Akishima, Tokyo, Japan). High-resolution TEM (HRTEM) was also conducted. Raman spectra were obtained using a XploRA PLUS confocal Raman spectrometer, (Horiba, Palaiseau, France). X-ray diffraction (XRD) patterns were collected using a D2 PHASER diffractometer, (Bruker, Bremen, Germany). X-ray photoelectron spectroscopy (XPS, K-Alpha, Thermo Fisher Scientific, Waltham, MA, USA) and ultraviolet photoelectron spectroscopy (UPS, Thermo ESCALAB 250Xi, Thermo Fisher Scientific, Waltham, MA, USA) were employed to analyze surface composition and electronic structures. Atomic force microscopy (AFM) images were recorded using an MFP-3D Infinity instrument, (Asylum Research (Oxford Instruments), Goleta, CA, USA). UV-vis diffuse reflectance spectra (DRS) were measured on a LAMBDA 750 spectrophotometer (PerkinElmer, Shelton, CT, USA). Photocurrent and electrochemical impedance spectroscopy (EIS) measurements were performed on a CHI 600E electrochemical workstation (Chenhua Instrument Co., Ltd., Shanghai, China). Photoluminescence (PL) and photoluminescence excitation (PLE) spectra were measured using an Edinburgh FLS1000 fluorescence spectrophotometer (Edinburgh Instruments Ltd., Livingston, UK). Time-dependent density functional theory (TD-DFT) calculations were carried out using Gaussian 09 software (Revision D.01), (Gaussian, Inc., Wallingford, CT, USA) at the B3LYP/6-31G(d,p) level to obtain the HOMO–LUMO energy levels and orbital distributions. Brunauer–Emmett–Teller (BET) surface areas and Barrett–Joyner–Halenda (BJH) pore size distributions were obtained from N_2_ adsorption–desorption isotherms recorded at 77 K on a Micromeritics ASAP 2460 surface area analyzer, Micromeritics Instrument Corp., (Norcross, GA, USA). Kelvin probe force microscopy (KPFM) measurements were performed using an MFP-3D Infinity atomic force microscope, (Oxford Instruments, Santa Barbara, CA, USA) to analyze the surface potential and charge separation behavior of the photocatalysts.

### 3.6. Photocatalytic Activity Tests

The initial methylene blue concentration (10 mg L^−1^) was chosen to ensure obvious concentration variation and accurate detection, suitable for kinetic evaluation. The photocatalytic performance was evaluated by the degradation of methylene blue (pH ≈ 6.8) under visible-light irradiation in a standard quartz photochemical reactor (Beijing Perfectlight Technology Co., Ltd., Beijing, China) (volume: 100 mL, equipped with a magnetic stirring device and a quartz window for light transmission). In a typical experiment, 20 mg of photocatalyst was dispersed in 50 mL MB solution (10 mg L^−1^). Prior to irradiation, the suspension was stirred in the dark for 30 min (500 rpm) to achieve adsorption-desorption equilibrium. During irradiation, the reaction temperature was controlled at 25 ± 1 °C by a circulating water thermostatic device (Shanghai Hengping Scientific Instrument Co., Ltd., Shanghai, China) attached to the photochemical reactor to exclude photothermal effects. A 300 W xenon lamp (CEL-HXF300-T3, Beijing China Education AuLight Co., Ltd., Beijing, China) was used as the light source and mounted 15 cm above the reactor to ensure uniform illumination. At 30 min intervals, 5 mL aliquots were withdrawn, centrifuged using a high-speed desktop centrifuge (Hunan Kaida Scientific Instrument Co., Ltd., Changsha, China), and analyzed using a UV-vis spectrophotometer (PerkinElmer Inc., Shelton, CT, USA) at 665 nm to determine MB concentration. Cycle tests were conducted to evaluate stability under identical conditions. A blank test without catalyst was performed to monitor direct MB photolysis. MB intermediates were identified by LC-MS (Agilent 1290-6530-QTOF, Agilent Technologies, Santa Clara, CA, USA).

## 4. Conclusions

Hierarchically porous hollow TiO_2_ nanofibers (HNFTi) and three types of GQD (b-, g-, o-GQDs) were successfully fabricated and combined to form heterojunction photocatalysts. The integration of GQDs enhances visible-light harvesting, narrows the bandgap, and effectively suppresses photogenerated carrier recombination. The progressive fluorescence redshift of GQDs further improves photocatalytic performance. Optimal loading of 0.5 wt% GQDs yielded the highest MB degradation efficiencies: 90.1% (0.5b-HNFTi), 94.3% (0.5g-HNFTi), and 99.5% (0.5o-HNFTi), such performance not only clearly outmatched those of the pristine TiO_2_ fibers but also was superior/comparable to most of the state-of-the-art photocatalyst counterparts (Table S2). The GQD/TiO_2_ heterojunctions effectively extend the light absorption range and promotes electron–hole separation, offering a promising strategy for designing high-performance photocatalysts for sustainable wastewater treatment. In future work, we plan to extend this photocatalytic system to treat other typical refractory pollutants in actual wastewater and explore its performance in continuous-flow reaction devices. In situ characterization and theoretical calculations will be further adopted to clarify the charge transfer mechanism. Meanwhile, large-scale preparation and long-term stability optimization will be carried out to promote the practical application of these high-performance heterojunction photocatalysts.

## Data Availability

The data can be provided upon request.

## Supplementary Materials

The following supporting information can be downloaded at: https://www.mdpi.com/article/10.3390/molecules31091430/s1, References [3,34,56,57,58,59,60,61] are cited in the Supplementary Materials.

## References

[1] Koe W.S., Lee J.W., Chong W.C., Pang Y.L., Sim L.C. (2020). An overview of photocatalytic degradation: Photocatalysts, mechanisms, and development of photocatalytic membrane. Environ. Sci. Pollut. Res..

[2] Hui K.C., Suhaimi H., Sambudi N.S. (2022). Electrospun-based TiO_2_ nanofibers for organic pollutant photodegradation: A comprehensive review. Rev. Chem. Eng..

[3] Safardoust-Hojaghan H., Salavati-Niasari M. (2017). Degradation of methylene blue as a pollutant with N-doped graphene quantum dot/titanium dioxide nanocomposite. J. Clean. Prod..

[4] Albayati T.M., Sabri A.A., Alazawi R.A. (2016). Separation of Methylene Blue as Pollutant of Water by SBA-15 in a Fixed-Bed Column. Arab. J. Sci. Eng..

[5] Feng D., Lü J., Guo S., Li J. (2021). Biochar enhanced the degradation of organic pollutants through a Fenton process using trace aqueous iron. J. Environ. Chem. Eng..

[6] Singh P., Borthakur A. (2018). A review on biodegradation and photocatalytic degradation of organic pollutants: A bibliometric and comparative analysis. J. Clean. Prod..

[7] Yang Y., Zhang C., Lai C., Zeng G., Huang D., Cheng M., Wang J., Chen F., Zhou C., Xiong W. (2018). BiOX (X = Cl, Br, I) photocatalytic nanomaterials: Applications for fuels and environmental management. Adv. Colloid Interface Sci..

[8] Che H., Che G., Jiang E., Liu C., Dong H., Li C. (2018). A novel Z-Scheme CdS/Bi_3_O_4_Cl heterostructure for photocatalytic degradation of antibiotics: Mineralization activity, degradation pathways and mechanism insight. J. Taiwan Inst. Chem. Eng..

[9] Li Y., Zhang P., Wan D., Xue C., Zhao J., Shao G. (2020). Direct evidence of 2D/1D heterojunction enhancement on photocatalytic activity through assembling MoS_2_ nanosheets onto super-long TiO_2_ nanofibers. Appl. Surf. Sci..

[10] Trevisan V., Olivo A., Pinna F., Signoretto M., Vindigni F., Cerrato G., Bianchi C.L. (2014). C-N/TiO_2_ photocatalysts: Effect of co-doping on the catalytic performance under visible light. Appl. Catal. B Environ..

[11] Li X., Wang C., Tang J. (2022). Methane transformation by photocatalysis. Nat. Rev. Mater..

[12] Guo Q., Zhou C., Ma Z., Yang X. (2019). Fundamentals of TiO_2_ Photocatalysis: Concepts, Mechanisms, and Challenges. Adv. Mater..

[13] Pasini S.M., Valério A., Yin G., Wang J., Souza S.M.A.G.U., Hotza D., Souza A.A.U. (2021). An overview on nanostructured TiO_2_-containing fibers for photocatalytic degradation of organic pollutants in wastewater treatment. J. Water Process Eng..

[14] Qian R., Zong H., Schneider J., Zhou G., Zhao T., Li Y., Yang J., Bahnemann D.W., Pan J.H. (2019). Charge carrier trapping, recombination and transfer during TiO_2_ photocatalysis: An overview. Catal. Today.

[15] Rawool S.A., Yadav K.K., Polshettiwar V. (2021). Defective TiO_2_ for photocatalytic CO_2_ conversion to fuels and chemicals. Chem. Sci..

[16] Chu S., Pan Y., Wang Y., Zhang H., Xiao R., Zou Z. (2020). Polyimide-based photocatalysts: Rational design for energy and environmental applications. J. Mater. Chem. A.

[17] Yin Z.-W., Betzler S.B., Sheng T., Zhang Q., Peng X., Shangguan J., Bustillo K.C., Li J.-T., Sun S.-G., Zheng H. (2019). Visualization of facet-dependent pseudo-photocatalytic behavior of TiO_2_ nanorods for water splitting using In situ liquid cell TEM. Nano Energy.

[18] Üstün B., Aydın H., Koç S.N., Kurtan Ü. (2023). Amorphous ZnO@S-doped carbon composite nanofiber for use in asymmetric supercapacitors. Diam. Relat. Mater..

[19] Kang M., Ruan Y., Lu Y., Luo L., Huang J., Zhang J.-M., Hong Z. (2019). An interlayer defect promoting the doping of the phosphate group into TiO_2_(B) nanowires with unusual structure properties towards ultra-fast and ultra-stable sodium storage. J. Mater. Chem. A.

[20] Tao Q., Wang B., Yan X., Chen L. (2019). Hybrids of TiO_2_ nanobelts modified by graphene: Preparation, characterization, and photocatalytic performance. Appl. Surf. Sci..

[21] Denisov N., Yoo J., Schmuki P. (2019). Effect of different hole scavengers on the photoelectrochemical properties and photocatalytic hydrogen evolution performance of pristine and Pt-decorated TiO_2_ nanotubes. Electrochimica Acta.

[22] Sidaraviciute R., Buivydiene D., Krugly E., Valatka E., Martuzevicius D. (2019). A composite microfibre-supported short-nanofibre photocatalyst for environmental pollutant decomposition. J. Photochem. Photobiol. A Chem..

[23] Qi L., Zhang H., Yang Y., Qi J., Zhou Y., Zhu Z., Li J. (2025). Coupled electron delocalization and multi-dimensional porosity engineering in TiO_2_ hollow spheres-embedded carbon nanofiber aerogels for efficient photocatalytic aniline degradation. Chem. Eng. J..

[24] Feng P., Dong K., Xu Y., Zhang X., Jia H., Prell H., Tovar M., Manke I., Liu F., Xiang H. (2024). Efficient and Homogenous Precipitation of Sulfur Within a 3D Electrospun Heterocatalytic Rutile/Anatase TiO_2_-x Framework in Lithium–Sulfur Batteries. Adv. Fiber Mater..

[25] Wisnet A., Bader K., Betzler S.B., Handloser M., Ehrenreich P., Pfadler T., Weickert J., Hartschuh A., Schmidt-Mende L., Scheu C. (2015). Defeating Loss Mechanisms in 1D TiO_2_-Based Hybrid Solar Cells. Adv. Funct. Mater..

[26] Wu F., Hu F., Hu P., Zhang P., Fan B., Kong H., Zheng W., Cai L., Sun Z. (2024). Multifunctional carbon Fiber@TiO_2_/C aerogels derived from MXene for pressure tunable microwave absorption. Carbon.

[27] Ye X., Ruan J., Pang Y., Yang J., Liu Y., Huang Y., Zheng S. (2021). Enabling a Stable Room-Temperature Sodium–Sulfur Battery Cathode by Building Heterostructures in Multichannel Carbon Fibers. ACS Nano.

[28] Covaliu-Mierlă C.I., Matei E., Stoian O., Covaliu L., Constandache A.-C., Iovu H., Paraschiv G. (2022). TiO_2_–Based Nanofibrous Membranes for Environmental Protection. Membranes.

[29] Zhang J., Hou X., Pang Z., Cai Y., Zhou H., Lv P., Wei Q. (2017). Fabrication of hierarchical TiO_2_ nanofibers by microemulsion electrospinning for photocatalysis applications. Ceram. Int..

[30] Tang Y., Hao R., Fu Y., Jiang Y., Zhang X., Pan Q., Jiang B. (2016). Carbon quantum dot/mixed crystal TiO_2_ composites via a hydrogenation process: An efficient photocatalyst for the hydrogen evolution reaction. RSC Adv..

[31] Zhang M., Wang F., Shi X., Wei J., Yan W., Dong Y., Hu H., Wei K. (2022). Preparation and photodegradation properties of carbon-nanofiber-based catalysts. Polymers.

[32] Du J., Wang H., Chen H., Yang M., Lu X., Guo H., Zhang Z., Shang T., Xu S., Li W. (2016). Synthesis and Enhanced Photocatalytic Activity of Black Porous Zr-doped TiO_2_ Monoliths. Nano.

[33] Choi Y., Kim H.-I., Moon G.-H., Jo S., Choi W. (2016). Boosting up the Low Catalytic Activity of Silver for H_2_ Production on Ag/TiO_2_ Photocatalyst: Thiocyanate as a Selective Modifier. ACS Catal..

[34] Rajender G., Kumar J., Giri P.K. (2018). Interfacial charge transfer in oxygen deficient TiO_2_-graphene quantum dot hybrid and its influence on the enhanced visible light photocatalysis. Appl. Catal. B Environ..

[35] Xiong Y., Li W., Zhang L., Li B., Li S., Feng Q., Liu J., Wang H., Chen D., Chen Y. (2025). Harnessing graphene quantum dots for the selective and sensitive detection of dye contaminants. Diam. Relat. Mater..

[36] Sun Y., Xue C., Chen L., Li Y., Guo S., Shen Y., Dong F., Shao G., Zhang P. (2021). Enhancement of interfacial charge transportation through construction of 2D-2D p-n heterojunctions in hierarchical 3D CNFs/MoS_2_/ZnIn_2_S_4_ composites to enable high-efficiency photocatalytic hydrogen evolution. Solar RRL.

[37] Zhang L., Luo W., Chen Y., Zheng J., Cao L., Duan L., Tang T., Wang Y. (2023). Green synthesis of boron-doped carbon dots from Chinese herbal residues for Fe^3+^ sensing, anti-counterfeiting, and photodegradation applications. J. Clean. Prod..

[38] Abdo D.M., Rasly M., Morsy M., El-Shazly A.N. (2025). Enhanced humidity-sensing performance with metallurgy-derived ZnO/GQDs nanocomposite. Diam. Relat. Mater..

[39] Guo H., Liu Z., Shen X., Wang L. (2022). One-Pot Synthesis of Orange Emissive Carbon Quantum Dots for All-Type High Color Rendering Index White Light-Emitting Diodes. ACS Sustain. Chem. Eng..

[40] Hu Q., Xu C., Hua Y., Qi C., Zhang J., Wang Y., Tang C. (2025). Construction of a novel point-layer structure BNNSs/GQDs composites and research on the regulation of its microwave absorption performance by N and F-doped GQDs. Diam. Relat. Mater..

[41] Li W., Zhang L., Jiang N., Chen Y., Gao J., Zhang J., Yang B., Liu J. (2022). Fabrication of Orange Fluorescent Boron-Doped Graphene Quantum Dots for Al^3+^ Ion Detection. Molecules..

[42] Nair R.V., Chandran P.R., Mohamed A.P., Pillai S. (2021). Sulphur-doped graphene quantum dot based fluorescent turn-on aptasensor for selective and ultrasensitive detection of omethoate. Anal. Chim. Acta..

[43] Kadian S., Manik G. (2020). Sulfur doped graphene quantum dots as a potential sensitive fluorescent probe for the detection of quercetin. Food Chem..

[44] Kalantari F., Amirmazlaghani M., Olyaee S. (2023). Enhanced UV-sensing properties by utilizing solution-processed GQD in GQDs/Porous Si heterojunction Near-UV photodetector. Mater. Sci. Semicond. Process..

[45] Ibarra-Prieto H.D., Garcia-Garcia A., Aguilera-Granja F., Navarro-Ibarra D.C., Rivero-Espejel I. (2023). One-Pot, Optimized Microwave-Assisted Synthesis of Difunctionalized and B–N Co-Doped Carbon Dots: Structural Characterization. Nanomaterials.

[46] Zhao X., Li J., Kong X., Li C., Lin B., Dong F., Yang G., Shao G., Xue C. (2022). Carbon dots mediated in situ confined growth of Bi clusters on g-C_3_N_4_ nanomeshes for boosting plasma-assisted photoreduction of CO_2_. Small..

[47] Wu Z., Pan T., Lin D., Xia W., Shan J., Cheng R., Yang M., Hu X., Nan K., Qi L. (2022). Biocompatible tumor-targeted GQDs nanocatalyst for chemodynamic tumor therapy. J. Mater. Chem. B.

[48] Hou W., Li Y., Wang K., Guo H., Zhang B., Wang L. (2025). Boosting photogenerated charge accumulation of oxidated carbon nitride nanotubes for efficient CO_2_ photoreduction. Sci. China Chem..

[49] Chen Y., Wang C., Chen J., Wang S., Ju J., Kang W. (2022). Preparing Biomass Carbon Fiber Derived from Waste Rabbit Hair as a Carrier of TiO_2_ for Photocatalytic Degradation of Methylene Blue. Polymers..

[50] He J., Zhao Y., Li Y., Yuan Q., Wu Y., Wang K., Sun K., Wu J., Jiang J., Zhang B. (2025). Joule Heating-Driven sp2-C Domains Modulation in Biomass Carbon for High-Performance Bifunctional Oxygen Electrocatalysis. Nano-Micro Lett..

[51] Li S., Li Y., Chen H., Yang Y., Lin Y., Xie T. (2022). N-Doped TiO_2_ Coupled with Manganese-Substituted Phosphomolybdic Acid Composites As Efficient Photocatalysis-Fenton Catalysts for the Degradation of Rhodamine B. Langmuir.

[52] An G., Wang K., Yang M., Zhang J., Zhong H., Wang L., Guo H. (2025). Rational Design of Metal-Free Nitrogen-Doped Carbon for Controllable Reduction of CO_2_ to Syngas. Molecules.

[53] Liu H., Du Y., Deng Y., Ye P.D. (2015). Semiconducting black phosphorus: Synthesis, transport properties and electronic applications. Chem. Soc. Rev..

[54] Wang K., Huang K., Wang Z., An G., Zhang M., Liu W., Fu S., Guo H., Zhang B., Lian C. (2025). Functional Group Engineering of Single-Walled Carbon Nanotubes for Anchoring Copper Nanoparticles Toward Selective CO_2_ Electroreduction to C_2_ Products. Small.

[55] Huang M., Li S., Zhang Z., Xiong X., Li X., Wu Y. (2017). Multifunctional high-performance van der Waals heterostructures. Nat. Nanotechnol..

[56] Tohamy H.A.S., Fathy N.A., El-Sakhawy M., Kamel S. (2023). Boosting the adsorption capacity and photocatalytic activity of activated carbon by graphene quantum dots and titanium dioxide. Diam. Relat. Mater..

[57] Heng Z.W., Chong W.C., Pang Y.L., Sim L.C., Koo C.H. (2022). Photocatalytic degradation of organic pollutants using green oil palm frond-derived carbon quantum dots/titanium dioxide as multifunctional photocatalysts under visible light radiation. Chin. J. Chem. Eng..

[58] Miao R., Luo Z., Zhong W., Chen S., Jiang T., Dutta B., Nasr Y., Zhang Y., Suib S.L. (2016). Mesoporous TiO_2_ modified with carbon quantum dots as a high-performance visible light photocatalyst. Appl. Catal. B Environ..

[59] Chinnusamy S., Kaur R., Bokare A., Erogbogbo F. (2018). Incorporation of graphene quantum dots to enhance photocatalytic properties of anatase TiO_2_. MRS Commun..

[60] Huo P., Zhao P., Shi X.B., Zhou Z.Y., Liu B. (2020). Enhanced photocatalytic performance of electrospun hollow titanium dioxide nanofibers decorated with graphene quantum dots. J. Mater. Sci..

[61] Hatefi R., Younesi H., Mashinchian-Moradi A. (2021). A facile decoration of anatase Fe_3_O_4_/TiO_2_ nanocomposite with graphene quantum dots: Synthesis, characterization, and photocatalytic activity. Adv. Powder Technol..

