# Supplementary material for: Hierarchically Porous Hollow TiO2 Nanofibers Coupled with Fluorescence-Tuned Graphene Quantum Dots for Efficient Visible-Light Photocatalysis
Source: Molecules. 2026 Apr 26;31(9):1430. doi: 10.3390/molecules31091430 (PMC13164789; doi:10.3390/molecules31091430)
Supplement: Supplementary file 1 [file molecules-31-01430-s001.zip › molecules-4263586-supplementary.pdf]

Supporting information

Hierarchically Porous Hollow TiO_2 Nanofibers Coupled with Fluorescence-Tuned Graphene Quantum Dots for Efficient Visible-Light Photocatalysis

Weitao Li,[a] *Zeyun Dong,*[a] *Zhengyu Zhang,*[b] *Luoman Zhang,*[a] *Qizhe Wang,*[a] *Shang Li,*[a] *Shuai Li,*[a] *Lei Wang,*[a,*] *Jialin Liu*[a,*]

[a] Textile and Garment Industry of Research Institute, Zhongyuan University of Technology, Zhengzhou 450007, P. R. China; liweitao@zut.edu.cn (W.L.); 2023117759@zut.edu.cn; z1543086543@163.com; wangqizhe@zut.edu.cn; lishang@zut.edu.cn; 2024117032@zut.edu.cn; 6765@zut.edu.cn; liujialin60761@163.com

[b] Department of Environment, Yangtze Delta Region Institute of Tsinghua University, Zhejiang, Jiaxing, 314006, China; zhangzyever@gmail.com

Experimental Section

1.1. Synthesis of TiO_2 nanowires (NWTi)

TiO_2 nanowires were synthesized via a hydrothermal method. In a typical procedure, anatase TiO_2 powder (1 g) was dispersed in 50 mL of NaOH solution (10 M) by ultrasonication for 1 h. The suspension was transferred into a Teflon-lined autoclave and heated at 180 °C for 48 h. After cooling, the product was repeatedly washed with deionized water until neutral pH was reached, followed by vacuum filtration. The resulting solid was calcined at 500 °C for 4 h (heating rate: 5 °C/min) to yield TiO_2 nanowires (denoted as NWTi).

1.2. Synthesis of conventional TiO_2 Nanofibers (NFTi)

TiO_2 nanofibers were prepared via electrospinning. PVP (2.4 g) and TBT (4 g) were dissolved in ethanol (20 mL) under vigorous stirring to obtain a clear precursor solution. Electrospinning was carried out under the following conditions: distance, 20 cm; voltage, +20 kV/–1.5 kV; and feed rate, 1 cm h^{-1}. After 4 h of spinning, the nanofiber mats were pre-oxidized at 200 °C for 2 h and subsequently calcined at 500 °C for 2 h to afford NFTi.

Supplementary Figures

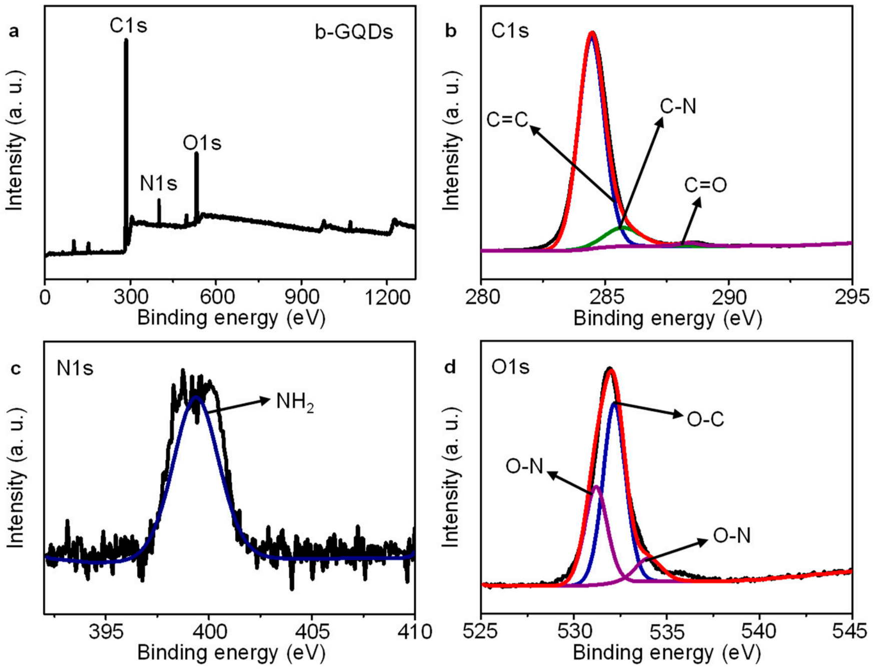

Figure S1 XPS patterns of b-GQDs.

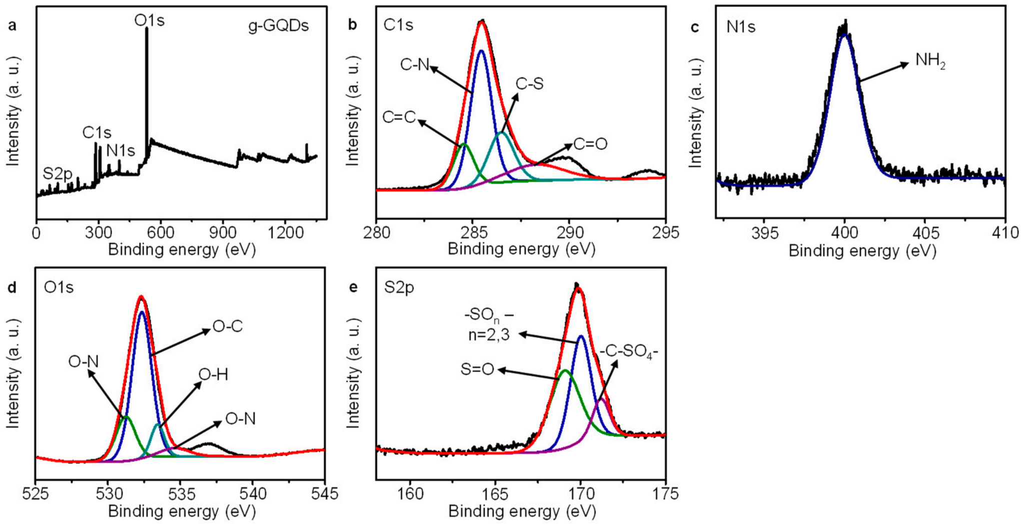

Figure S2 XPS patterns of g-GQDs.

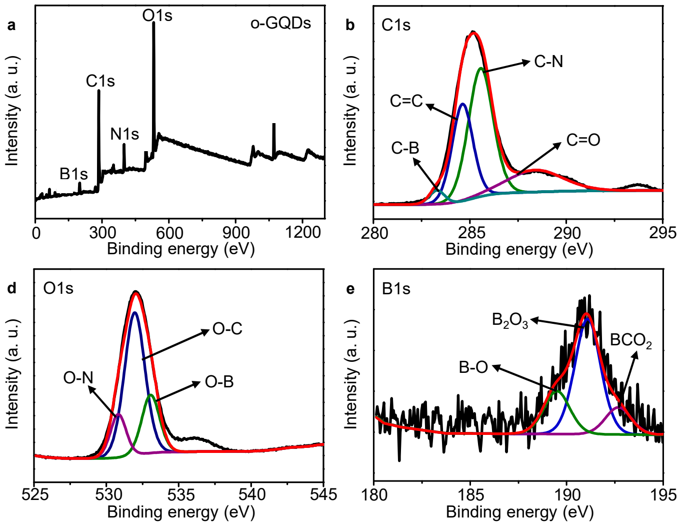

Figure S3 XPS patterns of o-GQDs.

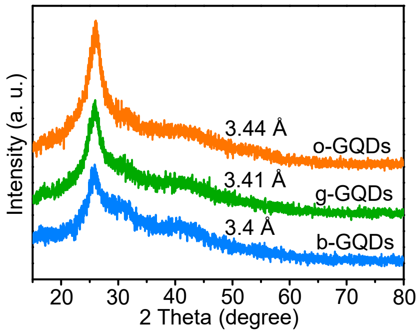

Figure S4 XRD spectra of b-, g-, and o-GQDs.

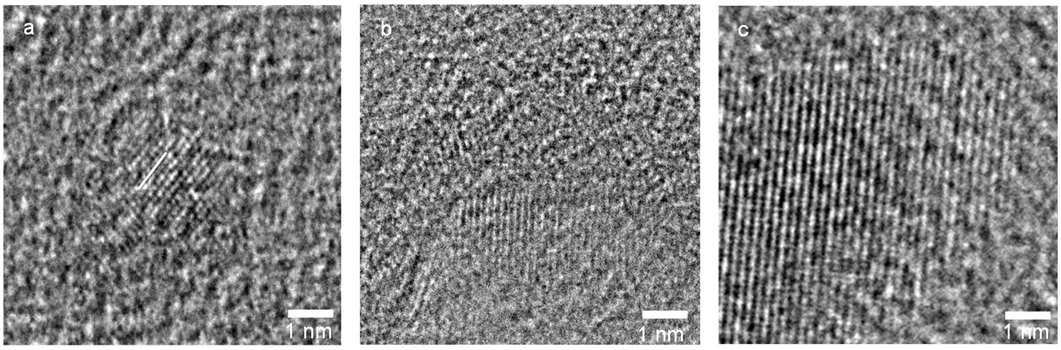

Figure S5 (a-c) SEM images of b-, g-, and o-GQDs.

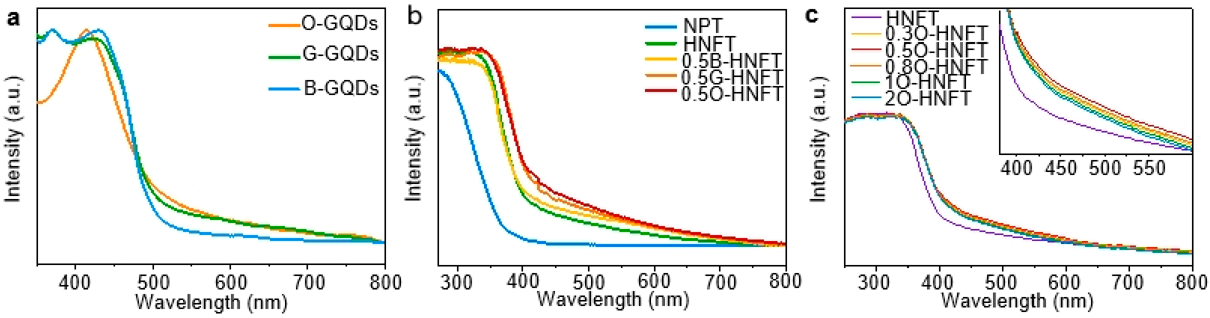

Figure S6 (a) UV–Vis DRS of b-, g-, and o-GQDs, (b) UV–Vis DRS of NPT,HNPT,0.5b-HNFT,0.5g-HNFT and 0.5o-HNFT,(c) UV–Vis DRS of HNFTs with different content of OGQDs

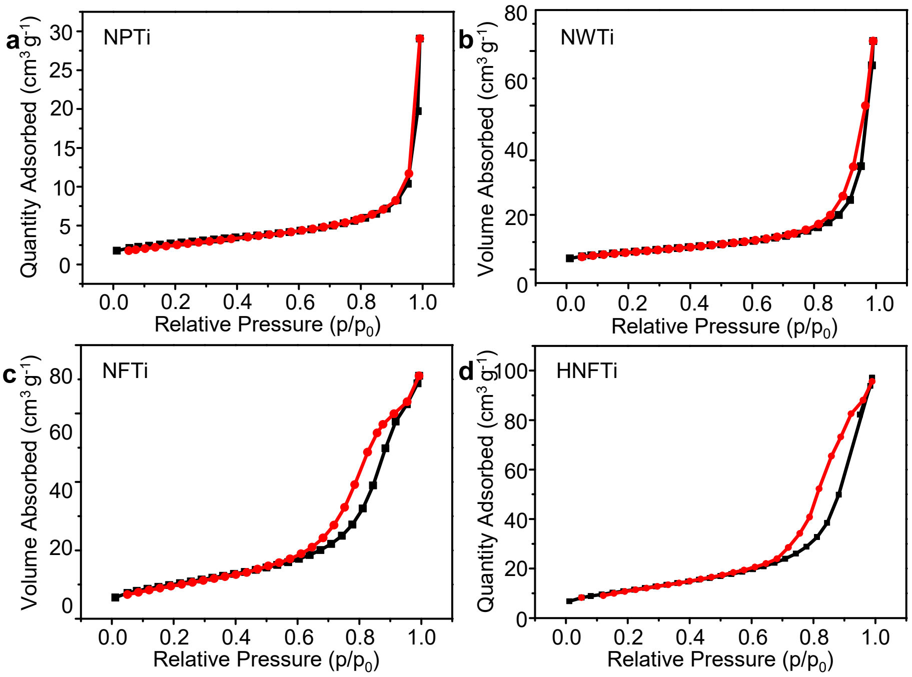

Figure S7 N_2 adsorption/desorption isotherms of (a) NPTi, (b) NWTi, (c) NFTi, and (d) HNFTi

Table S1 Energy gap, HOMO, and LUMO orbital energy levels for different GQDs.

**GQDs**	**λ_{edge}(nm)**	**HOMO (eV)**	**LUMO (eV)**	**E_{gopt} (eV)**
b-GQDs	525	-7.34	-4.98	2.2
g-GQDs	541	-7.53	-5.24	2.29
o-GQDs	563	-7.96	-5.76	2.36
HNFT		8.53	5.93	2.60

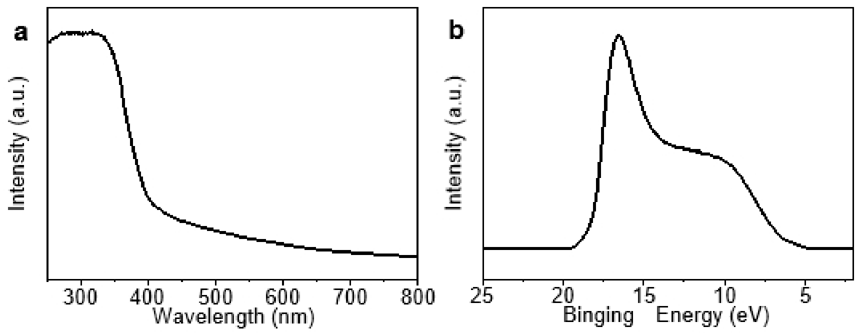

Figure. S8 (a-b) UPS of HNFT.

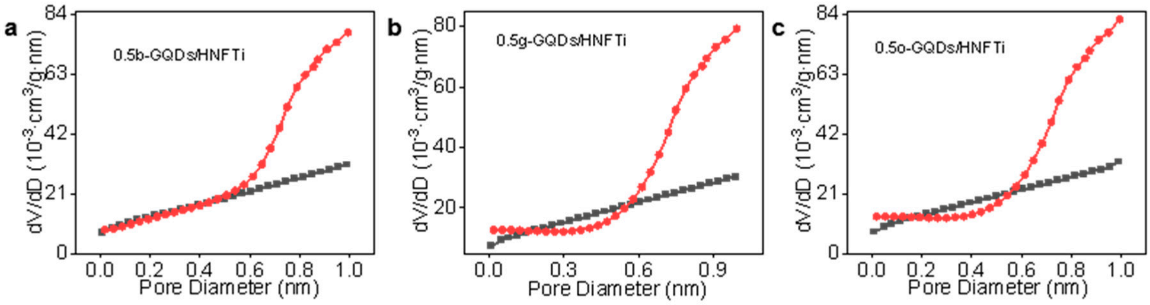

Figure S9 N_2 adsorption/desorption isotherms of (a) 0.5b-GQDs/HNFTi, 0.5g-GQDs/HNFTi,and 0.5o-GQDs/HNFTi

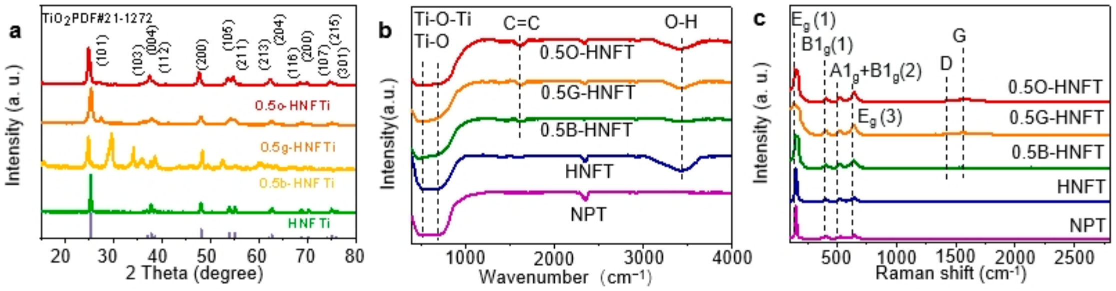

Figure S10 (a) XRD pattern of 0.5o-GQDs/HNFT, 0.5g-GQDs/HNFT, 0.5b-GQDs/HNFT and HNFT; (b) FT-IR spectra of 0.5o-, 0.5g-, 0.5b-HNFT and HNFT; (c) Raman spectra of0.5o-GQDs/HNFT, 0.5g-GQDs/HNFT, 0.5b-GQDs/HNFTHNFT and HNFT.

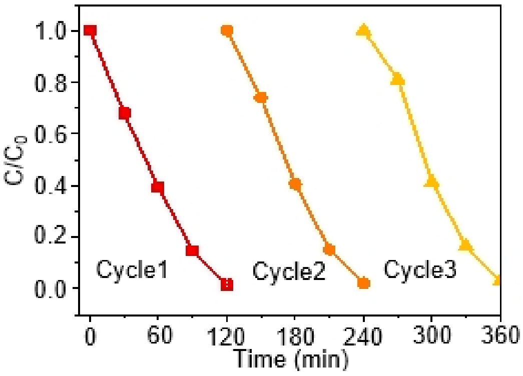

Figure S11 The degradation efficiency cycle diagram of MB.

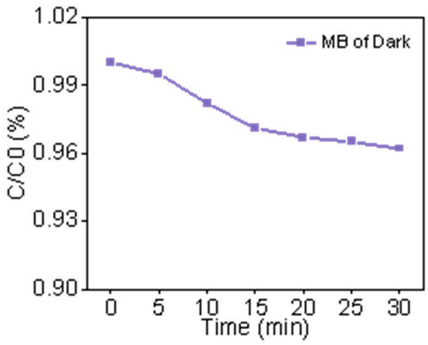

Figure S12 Dark-adsorption curve of MB over 0.5o-GQDs/HNFTi (C vs. t).

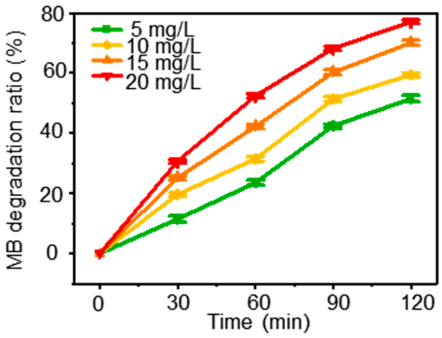

Figure S13 The degradation efficiency of MB by NPTi, NWTi, NFTi, and HNFTi under visible light is shown in the figure, which shows the degradation of MB for 2 h

Table S2 Comparison of photodegradation efficiency of MB by different composite photocatalysts.

Composite photocatalyst	pollutant concentration	Volume (mL)	Amount of photocatalyst	degradation efficiency	Light source type	references
GQDs-TiO_2/AC	MB: 400 mg/L	100 mL	100 mg	85.8%-97.6%	Visible light+0.01 mol H_2O_2	[1]
NCQDs/TiO_2	MB: 10 mg/L	100 mL	100 mg	86.16%	Visible light	[2]
CQDs/meso-Ti-450	MB: 32 mg/L	50 mL	50 mg	98%	Visible light	[3]
N-GQDs/TiO_2	MB: 10 mg/L	100 mL	100 mg	85%	UV-light	[4]
TiO_2-GQDs	MB: 10 mg/L	50 mL	50 mg	77%	UV-light	[5]
TiO_2-GQDs	MB:5 mg/L	100 mL	20 mg	99%	290-800 illumination	[6]
Fe_3O_4/TiO_2/GQDs	MB:10 mg/L	100 mL	500 mg	88.82±2.56%	UV-light	[7]
TiO_2-GQDs	MB: 8 mg/L	100 mL	20 mg	97%	Visible light	[8]

0.5o-HNFT	MB: 10 mg/L	50 mL	20 mg	99.5%	Visible light (λ＞420nm)	In this work

Reference

[1] Tohamy H-A S, Fathy N A, El-Sakhawy M, et al. Boosting the adsorption capacity and photocatalytic activity of activated carbon by graphene quantum dots and titanium dioxide. Diamond and Related Materials, 2023, 132: 109640.

[2] Heng Z W, Chong W C, Pang Y L, et al. Photocatalytic degradation of organic pollutants using green oil palm frond-derived carbon quantum dots/titanium dioxide as multifunctional photocatalysts under visible light radiation. Chinese Journal of Chemical Engineering, 2022, 51: 21-34.

[3] Miao R, Luo Z, Zhong W, et al. Mesoporous TiO_2 modified with carbon quantum dots as a high-performance visible light photocatalyst. Applied Catalysis B: Environmental, 2016, 189: 26-38.

[4] Safardoust-Hojaghan H, Salavati-Niasari M. Degradation of methylene blue as a pollutant with N-doped graphene quantum dot/titanium dioxide nanocomposite. Journal of Cleaner Production, 2017, 148: 31-36.

[5] Chinnusamy S, Kaur R, Bokare A, et al. Incorporation of graphene quantum dots to enhance photocatalytic properties of anatase TiO_2. MRS Communications, 2018, 8(1): 137-144.

[6] Huo P, Zhao P, Shi X, et al. Enhanced photocatalytic performance of electrospun hollow titanium dioxide nanofibers decorated with graphene quantum dots. Journal of Materials Science, 2020, 56(3): 2138-49.

[7] Hatefi R, Younesi H, Mashinchian-Moradi A, et al. A facile decoration of anatase Fe_3O_4/TiO_2 nanocomposite with graphene quantum dots: Synthesis, characterization, and photocatalytic activity[J]. Advanced Powder Technology, 2021, 32(7): 2410-22.

[8] Rajender G, Kumar J, Giri P K. Interfacial charge transfer in oxygen deficient TiO_2-graphene quantum dot hybrid and its influence on the enhanced visible light photocatalysis. Applied Catalysis B: Environmental, 2018, 224: 960-72.
